# Sexual dysfunction in Chinese rural patients with schizophrenia

**DOI:** 10.1186/s12888-019-2205-5

**Published:** 2019-07-12

**Authors:** Ying-Hua Huang, Cai-Lan Hou, C. H. Ng, Xie Chen, Qian-Wen Wang, Zhuo-Hui Huang, Fu-Jun Jia

**Affiliations:** 1 Shantou University Medical College, Shantou, Guangdong Province China; 2 Guangdong Provincial People’s Hospital, Guangdong Mental Health Center, Guangdong Academy of Medical Sciences, Guangzhou, Guangdong Province China; 3 Department of Psychiatry, The University of Melbourne, Melbourne, Victoria Australia; 4 The Second School of Clinical Medicine, Southern Medical University, Guangzhou, Guangdong Province China

**Keywords:** Sexual dysfunction, Quality of life, Schizophrenia, Rural

## Abstract

**Background:**

Sexual dysfunction is common in patients with schizophrenia, however it is poorly studied in China, especially in primary health care institutions in rural areas. We investigated the prevalence of sexual dysfunction and its correlates including quality of life (QoL), in schizophrenia patients treated in primary care in a rural area in China.

**Method:**

By using a random numbers table, 21 small town primary care service centers (from 63 totally) were selected in the study. Data of 720 community-dwelling patients with schizophrenia in rural area with diagnoses according to DSM –IV or ICD-10 were collected by interviews. Data on socio-demographic and clinical characteristics including sexual dysfunction and quality of life (QoL) were collected using a standardized protocol and data collection procedure. Data were analyzed using chi-square tests, t-tests, U-tests, ANCOVA and multiple logistic regression as appropriate by SPSS 21.0.The level of significance was set at 0.05 (two-tailed).

**Results:**

In this sample, sexual dysfunction was found in 71.3% of the whole sample, 82.7% of female patients and 64.5% of male patients. Multiple logistic regression analysis showed that older age (OR = 1.06, *P*<0.001, 95%CI: 1.04–1.09) and higher Brief Psychotic Rating Scale (negative domain) score (OR = 1.16, *P* = 0.01, 95%CI: 1.02–1.31) were significantly associated with sexual dysfunction. Contrary to previous findings, sexual dysfunction was not associated with quality of life after controlling for confounding variables.

**Conclusions:**

More than 2/3 of schizophrenia patients living in a rural area complained of sexual dysfunction, which was associated with older age and more negative psychotic symptoms. Primary care physicians should pay attention to sexual dysfunction during the assessment and treatment of patients with schizophrenia in rural areas in China.

## Background

Sexual dysfunction is common in patients with schizophrenia, which could be related to the psychotic illness itself, psychosocial factors, physical health, and the use of psychotropic medications [1–4], It has an impact on quality of life (QoL) and treatment adherence [5, 6], and some patients regard sexual dysfunction as a more severe problem than sedation, extrapyramidal or other side effects [7].

The prevalence of sexual dysfunction in patients with schizophrenia reported in studies from western countries varied widely, between 16 and 96% [8], but few such studies on schizophrenia patients have been done in rural areas. The large difference in prevalence of sexual dysfunction may derive from subject selection, assessment criteria of sexual function, or other sociocultural factors [9]. Gender, old age, the age of illness onset, psychopathology, antipsychotic use and side effects are commonly reported to be related to sexual dysfunction [10–14].

However, findings reported in western countries could not be generalized to other sociocultural contexts. For instance, schizophrenia patients living in Asia are less likely to report sexual dysfunction compared with their counterparts in western countries because of different attitudes to sexuality [15] and cultural sensitivities [16]. Being a sensitive issue in Asian countries, especially in rural areas, sexual function is given less attention by both patients and clinicians. Due to cultural barriers, schizophrenia patients may feel uncomfortable in raising their sexual issues and clinicians may be unwilling to discuss sexual problems, fearing that it may interfere with the doctor-patient relationship [12, 17].

The relationship between sexual dysfunction and quality of life reported in previous studies has been inconsistent. One study found that patients with sexual dysfunction had significantly poorer quality of life [18]. Similarly, another study found that sexual dysfunction was related to decreased subjective quality of life in patients with schizophrenia or schizoaffective disorder [19]. However, others reported no significant difference between sexual dysfunction and self-rated or observer-rated quality of life in schizophrenia patients [12].

Due to the lack of data especially in rural areas, and previous inconsistent findings, we therefore aimed to investigate the prevalence of sexual dysfunction in patients with schizophrenia treated by primary care physicians in rural areas in China, and explore its correlates with socio-demographic and clinical factors, and quality of life.

## Methods

### Study design and participants

This cross-sectional study using a standard protocol and data procedure was conducted by the Guangdong Mental Health Center of Guangdong Provincial People’s Hospital, cooperating with the Third People’s Hospital of Luoding. Luoding is located in a rural and undeveloped area, lying in the western side of Guangdong Province. The inclusion criteria were as follows: (1) meeting the diagnostic criteria of schizophrenia according to ICD − 10 via an interview by a clinical psychiatrist; (2) aged 18 years or older; (3) receiving treatment or mental health services from primary care physicians in community-based settings in Luoding; and (4) having the ability to read and understand the contents of the interview.

There are a total of 63 small town primary care services in Luoding, which is under the jurisdiction of Yunfu City. The survey sample size calculation took into account the estimated prevalence of sexual dysfunction (estimated as 40–80%), the absolute error (varying between 0.01 and 0.10), a confidence level of 95%, and the costs of data collection. The final target sample size was established at 750, which is about 30% of the total patients with schizophrenia in Luoding area. A stratified cluster was used. We randomly selected 21 small town primary care services in Luoding, using a random number table. All schizophrenic patients in the community treated in the primary health care services are registered in the Chinese National Psychiatric Management System. We tried to establish contact with all patients who are treated in the primary care services selected above by telephone at first. After explaining about the study, if the patients consented to participate, 3 trained psychiatrist went to the local primary health care institutions to make an interview with patients.

### Ethical consideration

Ethical clearance was obtained from the Ethics Committees of Guangdong Provincial People’s Hospital. After measuring the capacity of the patient to give consent, the trained interviews explained to each of the competent participant about the purpose, importance and confidentiality of the information gathered before we began the interview. Participants were also informed that they will not get any benefit because of participation in the study and no harm on them. Their willingness to be involved in the study was asked and written consent was obtained. Collectors followed the code of ethics and kept the participants’ privacy.

### Assessments

A standard research data form was used in the study by the researchers. Information on age, gender, education status, marital status, current drinking/smoking, age of illness onset, BMI, severe physical conditions, and family psychiatric history were collected. In addition, the use of medication including the first-generation antipsychotics (FGAs) and second-generation antipsychotics (SGAs), anticholinergics, antidepressants, and benzodiazepines were also recorded.

A common standard classification of stages in sexual response was used [20]. Sexual dysfunction was assessed by using the Arizona Sexual Experience Scale (ASEX) [21–23], which is a self-report questionnaire and has been validated in Chinese patients with schizophrenia with satisfactory psychometric properties [24]. ASEX quantifies sexual desire, arousal, vaginal lubrication/penile erection, ability to reach orgasm, and satisfaction with orgasm. The total score of ASEX is gained from the sum of the scores of five items with each score ranging from 1 to 6, so the total score range from 5 to 30. Higher scores indicate more serious sexual dysfunction. Sexual dysfunction is defined as a total score of ASEX ≥19 or any three ASEX items with a score ≥ 4 or any one item with an individual score ≥ 5 [22]. In this study, patients who have sexual partners are defined as having sexual dysfunction if their total score of ASEX ≥19. But patients without sexual partners only need to answer two items (strength of sex drive and ease of sexual arousal), and those who have any one item with a score ≥ 5 were defined as having sexual dysfunction.

The Brief Psychotic Rating Scale (BPRS) was used to estimate psychotic symptoms, according three domains: positive, negative, anxiety and tension [25, 26]. The Chinese version of the scale has good reliability and validity [25]. Depressive symptoms in the last week were assessed by using Chinese version of Montgomery-Asberg Depressive Rating Scale (MADRS) [26–28] which contains 10 items. Simpson and Angus Scale of Extrapyradimal Symptoms (SAS) was used to assessed the side effects including rigidity, akinesia, tremor and salivation of medicine [29]. The quality of life (QoL) was evaluated with the Chinese version of WHOQOL-BREF [30], which covers four domains: physical, psychological, social and environment. Higher scores of the QoL Scale indicate better quality of life.

The subjects in this study were recruited from Luoding city, an underdeveloped rural area in Guangdong, China. Prior to study commencement, three interviewers underwent an inter-rater reliability exercise on the use of the assessment scales in 20 schizophrenia patients. The inter-rater reliability of the rating instruments yielded excellent agreement (intra-class correlation coefficients and kappa values > 0.95).

### Statistical analysis

SPSS 21.0 for Windows was used to analyze the data. Chi-square tests were used to compare the difference of each domain of sexual dysfunction between males and females. T-test or Mann-Whitney U test was used to compare in demographic and clinical variables between patients with sexual dysfunction and without sexual dysfunction. Analysis of covariance was used to compare QoL between the above two groups after controlling for the potentially confounding effects of variables which differed significantly between groups. The association between demographic and clinical variables with sexual dysfunction was analyzed using the multiple logistic regression analysis. Sexual dysfunction was considered as the dependent variable, and the demographic and clinical characteristics which significantly differed (< 0.05) between the two groups were considered as independent variables, using ‘Enter’ method. Multicollinerity between independent variables was examined using variance inflation factor and correlation matrix. The level of significance was set at 0.05 (two-tailed).

## Results

### Socio-demographic characteristics

A total of 742 patients with schizophrenia from a rural area were enrolled in the study. Only 22 (2.0%) patients did not complete the interview or the ASEX survey. Altogether, 720 patients with schizophrenia were included in the final analysis. Among the participants 448 (62.2%) were male and 272 (37.8%) were female in gender. A total of 418 patients with schizophrenia who have sexual partners, while the other 302 patients did not have sexual partners.

Table 1 shows the frequency of different types of sexual dysfunction classified according to gender. Patients who were not sexually active only responded to two general sexual dysfunction domains. For this group, the prevalence of sexual dysfunction was 71.3% in the whole sample, 64.5% in males and 82.7% in females (X^2^ = 5.19, *P* <0.001). For sexually active patients who completed all five sexual domains, the prevalence of sexual dysfunction was 74.1% in the whole sample, and 67.8% in males, 82.1% in females (X^2^ = 3.36, *P* = 0.001). Female patients reported more sexual dysfunction both in terms of each sexual dysfunction domain or in the ASEX total score (*P* <0.05).

**Table 1:** Frequency of sexual dysfunction in patients with schizophrenia (Dysfunction≥5)

Whole Sample	Total (*n* = 720)		Male (*n* = 448)		Female (*n* = 272)		Statistics		
	*n*	%	*n*	%	*n*	%	χ2	df	*P*
ASEX Items									
Strength of sex drive	148	20.6	62	13.8	86	31.6	32.7	1	**<0.001**
Ease of sexual arousal	151	21.0	64	14.2	87	31.9	31.9	1	**<0.001**
Patients having sexual partners	Total (*n* = 418)		Male(*n* = 233)		Female(*n* = 185)		Statistics		
	*n*	%	*n*	%	*n*	%	χ2	df	*P*
Penile erection or vaginal lubrication	93	22.2	38	16.3	55	29.7	10.7	1	**0.001**
Ability to reach orgasm	95	22.7	39	16.7	56	30.2	10.7	1	**0.001**
Satisfaction of orgasm	94	22.5	38	16.3	56	30.2	11.5	1	**0.001**

Bold values: *P* < 0.05; *ASEX* Arizona Sexual Experience Scale

Table 2 shows the demographic and clinical characteristics in the sample who had sexual partners and in two groups separated into sexual dysfunction and non-sexual dysfunction. Among the participants with sexual partners, 310 (74.1%) had sexual dysfunction (ASEX ≥19).

**Table 2:** Comparison of basic demographic and clinical characteristics between patients with and without sexual dysfunction

	Sexual dysfunction (*n* = 310)		No Sexual Dysfunction (*n* = 108)		Statistics		
	*n*	%	*n*	%	X^2^	Df	*P*
Male Gender	158	50.9	75	69.4	11.08	1	**0.001**
Married	192	61.9	50	46.2	8.03	1	**0.005**
Employed	215	69.3	81	75	1.23	1	0.26
Living with others	287	92.5	106	98.1	4.41	1	**0.03**
Current smoker	38	12.2	17	15.7	4.14	2	0.12
Current drinker	11	3.5	9	8.3	4.02	1	**0.04**
First Episode	33	10.6	11	10.1	0.018	1	0.893
Physical condition	30	9.6	4	3.7	3.82	1	**0.05**
Psychiatric family history	45	14.5	11	10.1	1.20	1	0.27
Past physical violence	126	40.6	38	35.1	1.002	1	0.31
On antipsychotics							
On FGAs	91	29.3	31	28.7	0.016	1	0.89
On SGAs	139	44.8	57	52.7	2.02	1	0.15
Clozapine	59	19.0	21	19.4	0.009	1	0.92
SGAs except Clozapine	94	30.3	47	43.5	6.23	1	**0.01**
On antidepressants	5	1.6	1	0.9	0.002	1	0.96
On anticholinergics	102	32.9	32	29.6	0.39	1	0.53
	Mean	*SD*	Mean	*SD*	*T/Z*	df	*P*
Age	42.9	12.3	34.8	9.49	7.04	416	**<0.001**
Education	7.89	2.33	8.65	1.85	−3.42	416	**0.001**
Age Onset	27.9	10.1	24.4	6.2	4.16	416	**<0.001**
Illness Length	15.2	10.1	10.5	7.4	5.15	416	**<0.001**
BMI	21.50	2.60	22.09	2.50	−2.03	416	**0.043**
MARDS Total	4.77	5.78	3.44	4.85	2.33	416	**0.021**
BPRS Total	26.55	8.42	24.07	8.22	2.64	416	**0.008**
BPRS Positive	6.28	2.72	5.62	2.61	2.19	416	**0.029**
BPRS Negative	5.60	2.99	4.69	2.23	3.34	416	**0.001**
BPRS Anxiety	5.34	1.85	4.92	1.56	2.29	416	**0.02**
SAS Total	10.41	2.44	9.98	2.72	1.54	416	0.124
QOL Overall	11.92	2.23	12.22	2.19	−0.57	416	0.56
QOL Physical domain	12.21	1.48	12.34	1.38	−0.76	416	0.44
QOL Psychological domain	12.48	1.301	12.56	1.24	−0.59	416	0.54
QOL Social domain	12.09	1.91	12.209	2.04	−0.509	416	0.61
QOL Environment	11.94	1.40	12.01	1.05	−0.54	416	0.58

*BMI* Body Mass Index, *BPRS* Brief Psychiatric Rating Scale, *FGAs* first-generation antipsychotics, *MARDS* Montgomery-Asberg Depression Scale, *QOL* Quality of life, *SAS* Simpson and Angus Scale of Extrapyramidal Symptoms, *SGAs* second-generation antipsychotics

Bold value: *P* < 0.05

### Clinical characteristics

Table 2 shows that there was significant difference between sexual dysfunction group and non-sexual dysfunction group in BPRS total score(T = 2.64, *P* = 0.008), BPRS positive score (T = 2.19, *P* = 0.029), BPRS anxiety score (T = 2.29, *P* = 0.02),BPRS negative score (T = 3.34, *P* = 0.001), MADRS total score (T = 2.33, *P* = 0.021). However, there was no significant difference in side effect score (T = 1.54, *P* = 0.124).

### Quality of life

Concerning the quality of life, after controlling the variables which were significantly different between sexual dysfunction and non-sexual dysfunction groups, there was no significant difference in physical (F = 0.22, *P* = 0.63), psychological (F = 0.34, *P* = 0.55), social (F = 0.75, *P* = 0.38) or environment (*F* = 0.05, *P* = 0.82) domain.

Table 3 shows the independent demographic and clinical correlates of sexual dysfunction. Older age (aOR = 1.06, *P*<0.001, 95%CI: 1.04–1.09) and more BPRS negative symptoms (aOR = 1.16, *P* = 0.01, 95%CI:1.02–1.31) were significantly associated with sexual dysfunction in the binary regression analysis (Likelihood ratio:=410.53, R^2^ = 0.218).

**Table 3:** Independent social-demographic correlates of sexual dysfunction

	*P*	*OR*	95%*CI*
Age (years)	<0.001	1.06	1.02–1.09
Male gender	0.12	0.62	0.34–1.14
Married	0.59	0.85	0.47–1.52
Education (years)	0.36	0.94	0.84–1.06
Age Onset	0.74	1.007	0.96–1.05
BMI	0.17	0.93	0.83–1.03
Living with others	0.15	0.32	0.07–1.51
Medical condition	0.37	1.68	0.53–5.30
MARDS Total	0.82	0.99	0.93–1.06
BPRS Positive	0.48	0.95	0.84–1.08
BPRS Negative	0.01	1.16	1.02–1.31
BPRS Anxiety	0.07	1.20	0.98–1.47
SGAs except Clozapine	0.25	0.75	0.45–1.23

*BMI* Body mass index, *BPRS* Brief Psychiatric Rating Scale, *MARDS* Montgomery - Asberg Depression Scale, *SGA s* second-generation antipsychotics

A total of 302 patients with schizophrenia who did not have sexual partners participated in the study. These patients only answered first two items. There was a significant difference between strength of sex drive dysfunction group and strength of sex drive function group in gender (X^2^ = 24.3, *P*<0.001), marriage status (X^2^ = 21.9, *P*<0.001), with medical condition (X^2^ = 4.06, *P* = 0.04),age (T = -4.08, *P* = 0.001), ducation (T = 4.28, *P*<0.001), BPRS score (T = -2.92, *P* = 0.04), MADRS score (T = -2.35, *P* = 0.01).

There was a significant difference between ease of sexual arousal dysfunction group and ease of sexual arousal function group in gender (X^2^ = 23.1, *P*<0.001), marriage status (X^2^ = 16.1, *P*<0.001), with medical condition(X^2^ = 7.37, *P* = 0.007), age (T = -3.93, *P*<0.001),education (T = 3.80, *P*<0.001), BPRS score (T = -2.88, *P* = 0.004), MADRS score(T = -2.56, *P* = 0.01).

## Discussion

To our knowledge, this is the first study in China which investigated the frequency of sexual dysfunction and its associated factors in a sample of schizophrenia patients treated in primary care in a rural area.

The study found that the prevalence of sexual dysfunction is high in Chinese rural patients with schizophrenia, and they may need relevant intervention on this issue. Although the patients in this study treated in primary care are clinical stable, the prevalence of sexual dysfunction in patients with schizophrenia in our study is supported by some other studies in other countries. A study in Egypt on paranoid and non-paranoid schizophrenia patients reported that the prevalence of sexual dysfunction in paranoid schizophrenia patients reached 80% while the other group reached 86.7% [31]. A study conducted in Ethiopia in 2017 also reported high prevalence on sexual dysfunction in patients with schizophrenia, reaching 82.7% [32]. Contrary to these studies, the study conducted in Britain reported the prevalence of sexual dysfunction was 45% [33], nearly half of the prevalence of our study. The studies in Nigeria showed different results in different areas [34]. In another study conducted in Nigeria, the prevalence of sexual dysfunction with patients with psychotic disorder was higher in the northern region (64.3%) than in the southwestern region (40.4%) [34].

In general population, most studies have reported more common sexual dysfunction in females than in males [35, 36]. About 20–30% of men and 40–45% of women reported at least one item of sexual dysfunction [37–39]. In a previous study in India, 70% of female patients with schizophrenia reported having sexual dysfunction [40]. Our study found higher rates of total sexual dysfunction in female schizophrenia patients than male patients. But this difference in the rate of sexual dysfunction between genders is inconsistent in previous studies [11, 41, 42]. Some studies reported higher rates of sexual dysfunction in female patients [11, 34], others reported opposite results [41], while some found similar rates between genders [42].

The prevalence of sexual dysfunction was higher in females (82.7%), than in males (64.5%), which is consistent with findings reported in other studies (female 45–80% and male 30–80%) [1, 14, 17, 43]. Compared to our study conducted in the city areas of Guangdong using the same assessment tools which showed a prevalence of sexual dysfunction of 80.6% in females and 60.7% in males [44], the prevalence in the rural areas appear slightly higher than in city areas.

The discrepancies in the prevalence of sexual dysfunction in different areas and genders are due to multiple factors, including different study areas, sample size, socio-demographic factors, cultural environment (open or closed), assessment tools to assess sexual dysfunction (self-report or asked by researchers), gender preponderance, psychopathology and psychotropic medication use [14, 45]. As for the assessment tools, in a study in America, the researchers used the Global Impression of Sexual Function (GISF) to assess sexual dysfunction [46], while another study in Dallas of America used the Arizona Sexual Experience Scale (ASEX) to assess sexual dysfunction [21]. Different assessment tools may be one of the reason for the discrepancies between areas and genders. In addition, patient reports of sexual dysfunction are significantly influenced by cultural factors and social acceptance in discussing sexual issues. According to a meta analysis, the prevalence of sexual dysfunction reported in Asia countries was lower than western counties [15]. Further, clinicians themselves may be conservative in enquiring about sexual concerns or consider sexual-related complains as insignificant in schizophrenia patients as compared to positive psychotic symptoms [17].

Our study found a negative association between age and sexual dysfunction, which was consistent with our previous study in city areas [44]. Some studies showed that older age is a risk factor for sexual dysfunction not only in general population but also in schizophrenia patients [21, 35, 36, 47]. With increasing age and chronic course of illness, schizophrenia patients may suffer physical conditions such as hypertension, diabetes and others, which may influence sexual function adversely. However, a previous study found at age was not an independent risk factor of sexual dysfunction, although age and illness duration were negatively associated with sexual dysfunction in sexual desire, arousal and satisfaction domains [48].

In this study, we also found an association between negative symptoms and sexual dysfunction. Negative symptoms are core features of schizophrenia. In a case-control study, sexual dysfunction was associated with negative symptoms [11], while another schizophrenia study from Europe showed that negative symptoms predicted decreased libido [49].

Quality of life is increasingly used to assess treatment outcome for schizophrenia patients [12, 19, 50, 51], and sexual function is an important part of QoL, which could be influenced by social-demographic characteristics [19], psychopathology [12], treatment modalities (eg, antipsychotic drugs) [52–54] and side effects [15, 55]. One study reported that patients with schizophrenia were more dissatisfied with their sex life than any other aspects of life [56]. Sexual dysfunction can reduce the medication compliance [57], which can increase the risk of the illness recurrence, re-hospitalization [58] or suicide attempts [59], and lower the quality of life [59]. However, in contrast to the results reported in Ethopia,claiming that sexual dysfunction is highly associated with poor quality of life [32], we found no significant differences between the gender groups in any domains of QoL, which is consistent with our previous city study [44] . However, WHQoL is not specifically designed to measure the QoL for schizophrenia patients on sexual problems and only measures the QoL over last two weeks [30] rather than long term period.

The strength of this study is the sample was relatively homogeneous and was selected randomly from a rural area. However, there are several limitations in this study. Firstly, it was a cross-sectional design without control group from the general population and so the causality between sexual dysfunction and variables could not be assessed. Secondly, schizophrenia patients in this were clinically stable and living in the community and the results cannot be generalized to patients in other illness stages. Thirdly, we did not perform a follow-up sexual dysfunction assessment of the patients. Fourthly, ASEX is an evaluation tool rather than a diagnostic tool [60] and may be not sensitive enough to diagnose the sexual dysfunction.

## Conclusion

In conclusion, more than 2/3 patients with schizophrenia in the rural area reported sexual dysfunction, which was associated with older age and negative psychotic symptoms, but not with quality of life. Primary care physicians should pay attention to sexual dysfunction during the assessment and treatment of patients with schizophrenia in rural areas in China.

## Data Availability

The authors confirm that, for approved reasons, some access restrictions apply to the data underlying the findings. The data set contains identifying participant information, which is not suitable for public deposition. Data might be available upon request by contacting the corresponding author; however, the request must comply with confidentiality and ethics rules of the ethics committee of our institution.

## Abbreviations

ASEX: The Arizona Sexual Experience Scale
BMI: Body mass index
BPRS: The Brief Psychotic Rating Scale
FGAs: First-generation antipsychotics
MARDS: Montgomery-Asberg Depressive Rating Scale
QoL: Quality of life
SAS: Simpson and Angus Scale of Extrapyradimal Symptoms
SGAs: Second-generation antipsychotics
